# Registration of finger implants in the Dutch arthroplasty registry (LROI)

**DOI:** 10.1016/j.jpra.2024.05.006

**Published:** 2024-06-01

**Authors:** Esther van Santbrink, Antonius A. van den Hurk, Anneke Spekenbrink-Spooren, Juliette E. Hommes, Rutger M. Schols, Xavier H.A. Keuter

**Affiliations:** a Department of Plastic, Reconstructive, and Hand Surgery, Maastricht University Medical Center+, P. Debyelaan 25, 6229HX, Maastricht, the Netherlands; b Dutch Arthroplasty Register (LROI), ’s-Hertogenbosch, the Netherlands; c Department of Plastic, Reconstructive, and Hand Surgery, Isala Hospital, Zwolle, the Netherlands; d Department of Plastic, Reconstructive and Hand Surgery, Zuyderland Medical Center, Sittard-Geleen / Heerlen, the Netherlands; e Department of Plastic, Reconstructive and Aesthetic Surgery, University Hospital Brussels, Brussels, Belgium

**Keywords:** Finger arthroplasty, Impact factors, Arthroplasty registry, Orthopedic and plastic surgery, Observational early outcomes

## Abstract

Finger arthroplasty is commonly used to treat pain in the finger joints due to osteoarthritis or rheumatoid arthritis. Despite the procedure having existed for a relatively long time, it is still unknown which characteristics influence implant survival. The Dutch Arthroplasty Registry (LROI) is one of the 4 registries worldwide registering finger arthroplasties. This study aimed to investigate impact factors for implant survival regarding finger joint arthroplasty and assess registration completeness using the national healthcare claims database to compare.

A total of 951 primary arthroplasties and 84 revision arthroplasties of the finger joints were registered. A higher likelihood of primary and revision surgery was found in female patients. The third and fourth proximal interphalangeal (PIP) joints were the most frequently operated in primary surgery; however, the metacarpophalangeal (MCP) joints were the most frequently revised joints. Silicone implants were used in most cases and evenly throughout all digits. Suboptimal registration completeness was shown for plastic surgeons with just 35.5%–37.4% of all surgeries registered. Although orthopedic surgeons do not perform most surgeries on the hand, they registered 76.5%–78.2% of surgeries. No statistical analyses were justified, considering the low completeness and limited follow-up.

Female gender and PIP joint disease are possible risk factors for primary arthroplasty. MCP arthroplasties showed higher revision rates. However, participation rates and, therefore, data completeness were not optimal. To optimize participation, improving ease of registration should be explored. Furthermore, we urge readers who deal with joint implants to register their surgeries in the LROI database because only optimal registration completeness leads to high-quality data.

## Introduction

Arthroplasty of the finger joints is a widely applied procedure in hand surgery practice. This technique is used in the treatment of pain due to osteoarthritis, inflammatory arthritis, rheumatoid arthritis, or post-traumatic osteoarthritis.^1^, ^2^, ^3^ Arthroplasty can be performed in all finger joints, including the metacarpophalangeal (MCP), proximal interphalangeal (PIP), and distal interphalangeal (DIP) joints. The main goal of arthroplasty surgery is pain relief, which is commonly well achieved.^4^ However, previous research has shown that arthroplasty may lead to complications, such as dislocation, joint contracture, synovitis, and implant fracture or loosening, which can require revision surgery.^5^^,^^6^ Moreover, despite the joint replacement, finger functionality does not improve generally and may decrease in some cases due to scar tissue formation.^7^

There is no clear consensus regarding the best implant type.^8^ The implants, patient selection, and surgical techniques continuously evolve.^9^ Randomized studies and systematic reviews show different results in impact factors, such as age or implant type.^10^, ^11^, ^12^ However, larger studies describing implant use and impact factors are still lacking.^13^ A well-organized database in which implants are registered can provide large amounts of data for research and develop an evidence-based model for patient and implant selection. Moreover, this enables monitoring of implant performance regarding the development of implants. At the time of writing, this selection is mostly based on the prominence of the implant in the market and the preference of the surgeon. Research that correlates patient and clinical characteristics and outcomes with implant type can aid surgeons in decision-making. Moreover, it ensures that postmarket surveillance for medical implants is not under scrutiny of the vendor's selection bias.

The Dutch Arthroplasty Registry (LROI) is a general orthopedic arthroplasty quality registry that launched its finger implant registry in 2016.^14^ It is not the first finger implant registry worldwide—the Norwegian registry has more experience as it was launched in 1994.^15^ Similar to the Norwegian registry, the Swedish HAKIR registry also includes arthroplasties of the finger joints.^16^ Moreover, the United Kingdom has a registry for hand surgery called the UK Hand Registry (UKHR).^15^ However, to our knowledge, no public annual reports have been published. Other hand and wrist registries exist in Germany and Australia; however, these do not register arthroplasty of the finger joints.^15^ Registration in the registry for finger implants is performed mainly by plastic surgeons and orthopedic surgeons. For orthopedic surgeons, registration is monitored by their scientific organization, making registration at least semimandatory. For plastic surgeons and trauma surgeons, registration is completely voluntary. The LROI manages multiple databases for joint arthroplasty, mainly used by orthopedic surgeons. The Norwegian registry provides some data that shows a high-survival rate for MCP implants, with overall survival of 94%, 89%, 85%, and 84% after 5, 10, 15, and 20 years, respectively.^17^ To our knowledge, data on finger arthroplasties provided by the LROI have not yet been described in current literature.

This study aimed to investigate the potential of registries in finger arthroplasty by evaluating descriptive data regarding arthroplasty of the finger joints from 2017 to 2021, assessing its quality using registration completeness and describing possible impact factors.

## Methods

### Study design

All finger arthroplasties registered in LROI between 2017 and 2021 were selected. The LROI contains data on patient, prosthesis, and procedure characteristics of primary and revision arthroplasties. This includes gender, age, body mass index (BMI), smoking, side, surgical specialty, finger, joint, fixation, approach, survival days of implant, and revision characteristics, including type and reason for revision. Prosthesis characteristics are obtained from an implant library, in which characteristics are derived from article numbers of prosthesis components, as provided by the manufacturer. Surgeries are registered in the LROI by plastic, orthopedic, and trauma surgeons since finger arthroplasty is performed by all these specialties in the Netherlands.

### Participants

Patients were included if they were 18 years of age or older and had undergone a primary or revision arthroplasty procedure of the finger joints, registered in the LROI database between 2017 and 2021. Arthroplasty surgeries of the first carpometacarpal (CMC) joint were excluded because these arthroplasties use a different concept for implants, which will not be described in the current study. Arthroplasties of the interphalangeal joint of the thumb were included.

### Data management

The anonymized data of the registry are stored securely in the LROI facility. The subset of data described in this study is also stored in the private network of the plastic, reconstructive, and hand surgery department of the Maastricht University Medical Center. Access to the data is only provided to the research team.

### Assessment of registration completeness

The surgeries registered in the databases provided by the LROI will be compared with the surgeries that are reported in the Dutch health insurance claims database.^18^ This retrospective database reports all healthcare provided by all medical specialties in the Netherlands. The claims database only reports the frequency with which specific healthcare was provided. As health insurance is obligatory in the Netherlands, private healthcare is nonexistent for most treatments, decreasing the likelihood of selection bias. All registered joint arthroplasty procedures performed of the MCP, PIP, and DIP joints in the claims database were compared with those registered in the LROI database. The procedures are registered in the claims database using healthcare activities. All healthcare activities mentioning the use of implants of the finger joints were included in our analysis (see Table S1).

### Statistical analyses

Patient and clinical characteristics are presented as mean and standard deviation (±SD) for continuous variables and absolute amount and percentages for categorical variables. Kaplan–Meier analysis to assess survival of implants was not performed due to the suboptimal registration completeness which would lead to unreliable analyses.

The registration completeness was calculated as the percentage of registrations in the Dutch Arthroplasty Registry compared with the claims database. This provided a range of registration completeness since one of the health activities in the claims database described 2 or 3 procedures. Therefore, no exact number could be given.

SPSS IBM version 28 was utilized to manage the databases and perform statistical analyses.

## Results

### Patient and clinical characteristics

A total of 1020 MCP or interphalangeal joints were included in the LROI database. Of these joints, 951 had a primary arthroplasty registered in the LROI database. Of these primary arthroplasties, 688 implants were used in female patients (72%) and 263 in male patients (27%). The mean age was 65 years (SD ± 17 years). The mean BMI was 27 kg/m^2^ (SD ± 4.9 kg/m^2^). One hundred patients were registered as smokers (11%), whereas 754 patients did not smoke (79%). Furthermore, 428 implants were used in the left hand (45%) and 523 in the right hand (55%). Three hundred thirteen arthroplasties were performed on the middle finger (33%), 238 on the ring finger (25%), 219 on the index finger (23%), 119 on the little finger (13%), and 7 on the thumb (0.7%). Regarding specialty, 644 implants were registered by plastic surgeons (68%), 281 by orthopedic surgeons (30%), and 2 were registered by trauma surgeons (0.2%). Six hundred nineteen implants were registered of the PIP joint (65%), 255 of the MCP joint (27%), and 22 of the DIP joint (2.3%). Nine implants were cemented (0.6%). Regarding surgical technique, the most applied approach was the dorsal approach in 860 cases (90%). An overview of patient and clinical characteristics is presented in Table 1.

**Table 1:** Patient and clinical characteristics of primary surgery.

Characteristic	n= 951 (%)	Missing, n (%)
Gender		0
- Male	263 (28)	
- Female	688 (72)	
Age; Mean ± SD, years	65.0 ± 17.0	0
BMI; Mean ± SD, kg/m²	27.0 ± 4.9	126 (13)
Smoking		97 (10)
- Yes	100 (11)	
- No	754 (79)	
ASA classification		34 (3.6)
- I	189 (20)	
- II	549 (58)	
- III-IV	179 (19)	
Side		0
- Left	428 (45)	
- Right	523 (55)	
Finger		55 (5.8)
- Thumb	7 (0.7)	
- Index	219 (23)	
- Middle	313 (33)	
- Ring	238 (25)	
- Little	119 (13)	
Joint		55 (5.8)
- MCP	255 (27)	
- PIP	619 (65)	
- DIP	22 (2.3)	
Cemented	9 (0.9)	51 (5.4)
Approach		45 (4.7)
- Dorsal	860 (90)	
- Volar	38 (4.0)	
- Lateral	5 (0.5)	
- Other	3 (0.3)	
Specialism surgeon		24 (2.5)
- Plastic	644 (68)	
- Orthopedic	281 (30)	
- Trauma	2 (0.2)	
Revision (%)	15 (1.6)	0

SD, standard deviation; BMI, body mass index; ASA, American Society of Anesthesiologists; MCP, metacarpophalangeal; PIP, proximal interphalangeal; DIP, distal interphalangeal

### Diagnosis and indication

Overall, osteoarthritis was the most common indication for primary arthroplasty. In PIP and DIP arthroplasty, osteoarthritis was the indication in 82% and 86%, respectively. Osteoarthritis was the indication in 47% of cases affecting the MCP joint. Especially for the MCP joint, rheumatoid arthritis was relatively more common compared with other joints, with 43% of cases. For the PIP and DIP joints, the share of rheumatoid arthritis was 6.5% and 4.5%, respectively. Other less common diagnoses were post-traumatic, inflammatory arthritis, and osteonecrosis. A complete overview per joint is presented in Table S2.

### Operative details

We found silicone implants to be the most used implants for arthroplasty of all joints (78%), followed by pyrocarbon (4.6%). Other materials included cobalt chrome, titanium, and cobalt chrome with polyethylene. Data on implant material were missing in 137 cases. A complete overview per joint is presented in Table S3. Use of implant material was generally the same in all fingers. Silicone was used in all digits, varying from 72% in the third digit to 82% in the fourth digit. The thumb only included 7 implants, of which 4 materials were not known.

The index finger was operated on in 23% of all cases, of which 42% were MCP cases, 53% were PIP cases, and 4.6% were DIP cases. Regarding revisions, 32% were cases of the index finger. The total distribution of primary arthroplasties over the joints is presented in Table S4. We found PIP cases to be less common in the index finger (53%) compared with the other digits (69%–87%).

### Revisions

In the registry, 84 revision surgeries were registered. Fifty-five patients were female (65%), and 29 patients were male (35%). The mean age was 62.0 years (SD ± 10.4), and the mean BMI was 26.1 (SD ± 4.3). Nine patients were smokers (11%). Furthermore, 54 revisions were performed of the right hand (64%) and 30 of the left hand (36%). Forty-eight were performed by plastic surgeons (57%); 34 were performed by orthopedic surgeons (40%). Twenty-eight revisions were performed of the middle finger (33%), 27 of the index finger (32%), 14 of the ring finger (17%), 12 of the little finger (14%), and 1 of the thumb (1.2%). Forty-two revisions were performed of the MCP joint (50%) and 40 were of the PIP joint (48%). Only 15 revisions had a corresponding primary surgery registered, whereas 69 revisions did not. The mean time to revision for the 15 complete cases was 434 days (SD ± 277 days). See Table 2 for an overview.

**Table 2:** Patient and clinical characteristics of revision surgery.

Characteristic	n=84 (%)	Missing, n (%)
Gender		0
- Male	29 (35)	
- Female	55 (65)	
Age; Mean ± SD, years	62.0 ± 10.4	0
BMI; Mean ± SD, kg/m²	26.1 ± 4.3	16 (19)
Smoking		11 (13)
- Yes	9 (10.7)	
- No	64 (76.2)	
ASA classification		9 (11)
- I	11 (13)	
- II	44 (52)	
- III-IV	20 (24)	
Side		0
- Left	30 (36)	
- Right	54 (64)	
Finger		2 (2.4)
- Thumb	1 (1.2)	
- Index	27 (32)	
- Middle	28 (33)	
- Ring	14 (17)	
- Little	12 (14)	
Joint		2 (2.4)
- MCP	42 (50)	
- PIP	40 (48)	
Cemented	1 (1.2)	13 (15)
Specialism		2 (2.4)
- Plastic	48 (57)	
- Orthopedic	34 (40)	
Primary surgery registered	15 (18)	69 (82)

SD, standard deviation; BMI, body mass index; ASA, American Society of Anesthesiologists; MCP, metacarpophalangeal; PIP, proximal interphalangeal

Of all registered primary surgeries, the total revision percentage was 1.6%. When only including primary surgeries with a follow-up of at least 1 year, this revision percentage was 1.8%.

The most common reason for revision of the MCP joint was implant fracture (31%), followed by loose component (18%) and dislocation (14%). The most common reasons for revision of the PIP joint were implant fracture and dysfunction (14%), followed by instability (7.1%) and dislocation (6.0%). Other reasons for revision were bone resorption and osteophytes. A complete overview is presented in Table S5.

### Estimation of registration completeness

In the healthcare insurance claims database, between 2017 and 2021, a total of 2274 to 2380 surgeries containing finger implants of MCP or interphalangeal joints were registered. An overview per year is presented in Table 3. According to the claims database, plastic surgeons performed most surgeries (n = 1851–1947), followed by orthopedic surgeons (n = 403–412). Trauma surgeons were the least involved with these procedures (n = 20–21). The total amount of registered health activities had declined slightly from 2017 (n = 593–625) to 2021 (n = 408–429). Compared with the LROI database, this data suggest that registration completeness is 36%–37% for plastic surgeons, 76%–78% for orthopedic surgeons, and 9.5%–10% for trauma surgeons.

**Table 3:** Number of registered healthcare activities for arthroplasty with implants in MCP, PIP and DIP joint per specialism in the national claims database.

Specialism	2017	2018	2019	2020	2021	Total
Plastic Surgery (n)	490-517	394-419	335-348	302-313	330-350	1851-1947
Orthopedic surgery(n)	102-107	109-111	68-69	51	73-74	403-412
Trauma surgery(n)	1	5	5-6	4	5	20-21
Total(n)	593-625	508-535	408-423	357-368	408-429	2274-2380

## Discussion

This study presents insights into the use and registration of finger implants in primary and revision arthroplasty and shows potential impacting factors for primary surgery and revision. To our knowledge, this is the first study in the Netherlands using finger arthroplasty data from the Dutch Arthroplasty Registry.

We found higher primary operation rates in women, which is consistent with findings by Notermans et al.^19^ and Brendsdalet al.^17^ This may be explained by osteoarthritis being the most common diagnosis, which is more prevalent in postmenopausal female patients.^20^ Patient characteristics for the revised implants are generally the same as the baseline characteristics. The age in the revised implants group was slightly lower. This could be a result of a more active and demanding patient group leading to more mechanical stress on the implant.^10^ These findings are consistent with the results from the studies conducted by Brendsdal et al.^17^ and Notermans et al.^19^ associating a younger age with higher revision rates in MCP implants.

Arthroplasty of any joint is slightly more prominent in the right hand, which comprised 55% of primary arthroplasty and 64% of revision cases. Revisions seem to be more common in the right hand, with primary surgeries being relatively equally distributed. Since 90% of the general population is right-handed,^21^ this might also occur due to excess mechanical stress.

While osteoarthritis is the most registered indication for all joints, rheumatoid arthritis is a relatively more common indication for primary arthroplasty of the MCP joint. This could be related to the known failure of joint gliding of the MCP joints in rheumatoid arthritis, leading to erosion of the proximal phalanx joint surface.^22^ Additionally, the bone-to-bone contact force in the MCP joints tends to be higher than in the interphalangeal joints.^23^ This variation is consistent with the data presented in the annual report of the Norwegian Registry.^24^ Furthermore, revision was more common in the MCP joint; this might also be attributable to the higher forces in the MCP joints, which may lead to implant-related failure, as seen in our study.

In the current literature, the index finger is described as less suited for arthroplasty because of a high-coronal load during lateral pinching, which may cause instability.^7^ In our study, we found 23% of primary operations to have been performed on the index finger and 30% of revisions. We also observed a relatively lower percentage of PIP cases in the index finger, which might be caused by patient selection since the index finger is deemed as being more susceptible to complications.

Silicone was the most common implant material for all joints. Surgeons’ preferences and experience may play a role. Chan et al.^11^ and Forster et al.^25^ show a lower rate of revision when using a silicone implant compared with pyrocarbon and metal-polyethylene. However, recently, newer implants have been introduced, such as the recent introduction of CapFlex implants with their respective 5-year follow-up.^26^ Although postmarketing studies on the performance of implants exist, a registry could provide independent insights into the use in practice and, therefore, will provide value in implant selection.

We identified a revision rate of 1.6% in the registered primary surgeries, which is much lower than the findings of Pritsch and Rizzo,^27^ and Notermans et al.^28^. This might be attributable to the continuous development and improvement of implants. However, inaccuracy might occur when surgeons do not report revision or removal of an implant. Furthermore, the follow-up was limited, especially for the more recently registered implants. Excluding primary surgeries with a follow-up of less than 1 year from this calculation did not affect the revision rate in a major way.

Patient-reported outcome measures (PROMs) are not used in this study because they are not yet available for finger implants in the LROI. These PROMs could give a more detailed insight into the practical performance of an implant since a discrepancy between objective and subjective outcomes has been observed in finger arthroplasty.^28^^,^^29^ Although registration of PROMs comes with large amounts of data flow and substantial costs (for patients, LROI, and healthcare providers), its viability should be considered.

Since registries are independent and of a larger scale than cohort studies or post market surveillance, the authors strongly believe that implant registries provide a great deal of independent data on implant use and survival. Moreover, the LROI database is a joint database, which is a large upside in the field of finger arthroplasty care regarding multidisciplinary character with multiple specialties performing similar surgeries. However, to perform quality control and benchmarking, sufficient data is necessary. As described, this study found suboptimal registration completeness, which varied per specialism. Orthopedic surgeons registered an estimated 76%–78% of all implants, compared with 36%–37% registered by plastic surgeons and 9.5%–10% by trauma surgeons. The monitored registration by the scientific association applies only to orthopedic surgeons in the Netherlands. For plastic surgeons and trauma surgeons, registration in the LROI database is completely voluntary. Moreover, the LROI is widely used over various arthroplasties in orthopedic surgery. Therefore, orthopedic surgeons are much more aware of its existence than plastic surgeons, whose only interaction with the LROI would be over hand and wrist implants. These points partially explain the discrepancy between the specialties and suggest that mandatory registration could contribute to better registration completeness. As an example, the Dutch Breast Implant Registry (DBIR) employs mandatory registration, and completeness is over 93% in all datapoints.^30^

When looking at the annual report of the LROI, registration completeness of 84% is reported for plastic surgeons and 81% for orthopedic surgeons regarding primary operations.^14^ This completeness is much higher than our calculations. This may be a result of the different methods for assessment of completeness. The LROI receives the control data from the different treatment centers. Our calculations used data directly from the financial billing database, which is thought to be more accurate. Our method of calculation has also been used to verify data completeness of the DBIR.^31^ To perfectly observe the completeness, the LROI database should be linked to the claims database using patient-specific information. However, ethical dilemmas could then occur.

At this time, due to the suboptimal registration completeness, limited number of complete cases, and limited follow-up in the current database, statistical analyses were not performed. To create a functioning registry that enables more research based on the registration of finger arthroplasties in the Netherlands, better registration is essential. Our study shows that registration completeness, and thereby data quality, is far from optimal. Therefore, the main takeaway of the current study should be the need for improved registration. To improve registration, 2 actionable points stand out. First, more awareness should be raised concerning registration, especially since both specialties are already familiar with implant registration of other surgeries. Second, the administrative burden should be minimized, making registration accessible and straightforward. Because an administrative burden is inherently connected to a registry, it may be beneficial to explore options to combine the registry with the financial database. Probably, the only way to achieve the highest registration completeness is to strive for obligatory registration monitored by the scientific associations. International collaboration might be of great value in assessing implant performance and improving patient selection if national databases register comparable data. However, in every scenario, the accuracy of the registration should be the main concern. Since large-scale registries are prone to produce lower-quality data, future modifications to the registration process should only be made if the quality of the database is ensured.

This study has various limitations. First, the registration completeness was suboptimal. This resulted in insufficient data for analysis. Second, the data in the registry are susceptible to errors because it is manually registered. Third, the data presented in the study only show short-term results. The limited follow-up time in this study might lead to underestimating the revision rates. Fourth, PROMs were not used. Finally, the data in the claims database are anonymized, and, therefore, the completeness of this database cannot be perfectly assessed. Moreover, healthcare provided abroad might have been declared in the Netherlands and, therefore, included in the database. We contacted the Dutch healthcare authority to assess the scope of this limitation. However, this was not possible since no registration on this subject was performed.

## Conclusions

Female gender and PIP joint disease are possible risk factors for primary arthroplasty. MCP arthroplasties show higher revision rates. However, the national participation rate for registration is not yet optimal. National registries, such as the LROI, can provide long-term follow-up data to help understand the current and future application of finger implants, thereby improving finger implant surgery and patient safety. In parallel, improving the ease of registration should be explored. We would like to urge readers who deal with implant surgery in their practice to consider registration of the implants using applicable registries to optimize registration completeness.

## Conflict of interest statement

AS is employed by the LROI as a researcher. All remaining authors have declared no conflicts of interest.

## Appendix Supplementary materials

Image, application 1

Image, application 2

Image, application 3

Image, application 4

Image, application 5
