# Supplementary material for: Registration of finger implants in the Dutch arthroplasty registry (LROI)
Source: JPRAS Open. 2024 Jun 1;41:215–24. doi: 10.1016/j.jpra.2024.05.006 (PMC11266863; doi:10.1016/j.jpra.2024.05.006)
Supplement: Supplementary file 1 [file mmc1.docx]

*Table S1. Healthcare activity; reference number and definition following the Dutch Health Authority (NZA) in 2021*

| Reference number | Definition |
| --- | --- |
| 38308 | Implant usage in a metacarpal-phalangeal joint |
| 38302 | Implant removal from a finger or hand, and reimplanting a new implant |
| 38306 | Implant usage in an interphalangeal joint |
| 38313 | Implant removal from a metacarpal-phalangeal joint |
| 38311 | Implant removal from a finger or hand |
| 38384 | Arthroplasty of a metacarpal-phalangeal joint or an interphalangeal joint, including implant |
| 38304 | Implant removal from a metacarpal-phalangeal joint, and reimplanting a new implant |
| 38381 | Arthroplasty of two or three metacarpal-phalangeal joints, including synovectomy and extensor tendon reimplantation. |
