# Supplementary material for: Registration of finger implants in the Dutch arthroplasty registry (LROI)
Source: JPRAS Open. 2024 Jun 1;41:215–24. doi: 10.1016/j.jpra.2024.05.006 (PMC11266863; doi:10.1016/j.jpra.2024.05.006)
Supplement: Supplementary file 2 [file mmc2.docx]

*Table S2: Indication for primary surgery per joint.*

|  | MCP | PIP | DIP |
| --- | --- | --- | --- |
| Osteoarthritis (%) | 120 (47) | 509 (82) | 19 (86) |
| Rheumatoid arthritis (%) | 110 (43) | 40 (6.5) | 1 (4.5) |
| Posttraumatic (%) | 6 (2.4) | 40 (6.5) | 1 (4.5) |
| Inflammatory arthritis (%) | 6 (2.4) | 7 (1.1) | 0 |
| Osteonecrosis (%) | 3 (1.2) | 0 | 0 |
| Other (%) | 6 (2.4) | 9 (1.5) | 0 |
| Missing (%) | 4 (1.6) | 14 (2.3) | 1 (4.5) |
