# Supplementary material for: Registration of finger implants in the Dutch arthroplasty registry (LROI)
Source: JPRAS Open. 2024 Jun 1;41:215–24. doi: 10.1016/j.jpra.2024.05.006 (PMC11266863; doi:10.1016/j.jpra.2024.05.006)
Supplement: Supplementary file 3 [file mmc3.docx]

*Table S3: Materials used in primary surgery per joint.*

|  | MCP | PIP | DIP |
| --- | --- | --- | --- |
| Silicone (%) | 189 (74) | 493 (80) | 14 (64) |
| Pyrocarbon (%) | 9 (3.5) | 32 (5.2) | 0 |
| Cobalt chrome (%) | 0 | 4 (0.6) | 0 |
| Titanium (%) | 1 (0.4) | 3 (0.5) | 0 |
| Cobalt chrome + polyethylene (%) | 4 (1.6) | 10 (1.6) | 0 |
| Unknown (%) | 52 (20) | 77 (12) | 8 (36) |
