# Supplementary material for: Registration of finger implants in the Dutch arthroplasty registry (LROI)
Source: JPRAS Open. 2024 Jun 1;41:215–24. doi: 10.1016/j.jpra.2024.05.006 (PMC11266863; doi:10.1016/j.jpra.2024.05.006)
Supplement: Supplementary file 4 [file mmc4.docx]

*Table S4: Distribution of primary arthroplasties over all joints per digit.*

| **Digit** | **MCP** | **PIP** | **DIP** |
| --- | --- | --- | --- |
| **First** | 4 (57%) | *N/A* | 3 (43%) |
| **Second** | 93 (42%) | 116 (53%) | 10 (4.6%) |
| **Third** | 96 (31%) | 215 (69%) | 2 (0.6%) |
| **Fourth** | 29 (12%) | 206 (87%) | 3 (1.3%) |
| **Fifth** | 33 (28%) | 82 (69%) | 4 (3.4%) |
