# Supplementary material for: Registration of finger implants in the Dutch arthroplasty registry (LROI)
Source: JPRAS Open. 2024 Jun 1;41:215–24. doi: 10.1016/j.jpra.2024.05.006 (PMC11266863; doi:10.1016/j.jpra.2024.05.006)
Supplement: Supplementary file 5 [file mmc5.docx]

*Table S5: Reason for revision.*

|  | MCP (n=42) | PIP (n=40) |
| --- | --- | --- |
| Infection (%) | 1 (2.4%) | 0 |
| Implant fracture (%) | 26 (62%) | 12 (30%) |
| Instability (%) | 11 (26%) | 10 (25%) |
| Dislocation (%) | 12 (29%) | 6 (15%) |
| Loose component (%) | 15 (36%) | 1 (2.5%) |
| Bone resorption (%) | 6 (14%) | 1 (2.5%) |
| Dysfunction (%) | 0 | 12 (30%) |
| Osteophytes (%) | 0 | 5 (13%) |
